# Nicotine and periodontal tissues

**DOI:** 10.4103/0972-124X.65442

**Published:** 2010

**Authors:** Ranjan Malhotra, Anoop Kapoor, Vishakha Grover, Sumit Kaushal

**Affiliations:** *Department of Periodontology and Oral Implantology, National Dental College & Hospital, Derabassi, Punjab, India*

**Keywords:** Nicotine, smoking, smoking cessation, tobacco

## Abstract

Tobacco use has been recognized to be a significant risk factor for the development and progression of periodontal disease. Its use is associated with increased pocket depths, loss of periodontal attachment, alveolar bone and a higher rate of tooth loss. Nicotine, a major component and most pharmacologically active agent in tobacco is likely to be a significant contributing factor for the exacerbation of periodontal diseases. Available literature suggests that nicotine affects gingival blood flow, cytokine production, neutrophil and other immune cell function; connective tissue turnover, which can be the possible mechanisms responsible for overall effects of tobacco on periodontal tissues. Inclusion of tobacco cessation as a part of periodontal therapy encourages dental professionals to become more active in tobacco cessation counseling. This will have far reaching positive effects on our patients’ oral and general health.

## INTRODUCTION

Tobacco use in any form has the potential to profoundly alter the systemic and oral health of the individual. The use of tobacco is associated with a wide spectrum of disease including stroke, coronary artery disease, peripheral artery disease, gastric ulcer and cancers of mouth, larynx, esophagus, pancreas, bladder and uterine cervix. It is also a major cause of chronic obstructive pulmonary disease and risk factor for low birth weight babies.[1] It has also been recognized to be a significant risk factor for periodontitis affecting the prevalence, extent and severity of disease. Tobacco use is associated with increased pocket depths, loss of periodontal attachment, alveolar bone and higher rate of tooth loss. In addition, it may influence the clinical outcome of non surgical and surgical therapy as well as long term success of implant placement.[2]

## ADVERSE HEALTH EFFECTS OF TOBACCO USE

### Systemic effects of tobacco use[3]

Cancer, cardiovascular disease, hypertension, stroke, respiratory disease, reproductive problems, impotence, ulcers, osteoporosis, facial wrinkling and nicotine addiction.

### Oral effects of tobacco use[3]

Cancers of mouth, pharynx, larynx, esophagus and lip, tooth abrasion, stain, calculus build up, halitosis, impaired taste and smell, attrition, delayed wound healing/dry socket, hairy or coated tongue, increased risk of cleft palate/lip, increased risk of tooth anomalies and/or growth retardation in newborns of mothers who smoke, leukoedema, nicotine stomotitis, periimplantitis, pre cancerous lesions including leukoplakia, erythroplakia, dysplasia, hyperkeratosis, periodontal disease/ gingival recession, sinusitis, tooth loss and xerostomia.

## TOBACCO-INDUCED PERIODONTAL TISSUE CHANGES

Pindborg (1947) was one of the first investigators to study the relationship between smoking and periodontal disease. He discovered a higher prevalence of acute necrotizing ulcerative gingivitis. Early studies show that smokers had higher levels of periodontitis but they also had poorer levels of oral hygiene and higher levels of calculus [Table 1].

**Table 1:** Tobacco induced periodontal tissue changes

Tissue changes with use	Biologic bases for changes	Tissue changes with abstinence
Paler tissue color	Increased vasoconstriction	Increased blood flow
Decreased bleeding	Oxygen depletion	Initially more bleeding, erythema
Thickened fibrotic consistency, minimal erythema relative to extent of disease	Compromised immune response:	Healthier consistency and anatomy
	Fewer and impaired PMNS	
	Reduced IgG antibody	
Gingival recession around anterior sextants	Increased collagenase production	
Greater probing depth, bone and attachment loss, furcation invasion	Reduction of bone mineral, impaired fibroblast formation	Stabilization of attachment levels
Refractory status	Impaired wound healing	

This paper reviews the potential impact of both smoking and smokeless tobacco use on periodontal disease.

### 1. Effects of smoking on periodontal disease

The relationship between smoking and periodontal diseases has been studied extensively over the past 15 years and both cross-sectional and longitudinal studies provide strong epidemiologic evidence of a positive association between smoking and clinical and radiographic signs of periodontitis, as well as an increased risk of periodontitis in smokers,[4–11] In a 10-year longitudinal radiographic study of alveolar bone loss, smoking was a significant predictor of future bone loss in those subjects who had at least 20 teeth at the beginning of the study.[12] In a five-year study of attachment loss in 800 community dwelling adults, smokers were found to be at an increased risk of attachment loss. In a further 12-month longitudinal study, smokers were shown to be at significantly greater risk for attachment loss than nonsmokers, the odds ratio being quoted as 5.4.[13] In one of the largest studies of risk factors for periodontal disease with 1361 subjects from Erie County, NY, aged 25–74 years; it was shown that smokers were at greater risk of experiencing severe bone loss than nonsmokers, with odds ratios ranging from 3.25 to 7.28 for light and heavy smokers, respectively.[14]

A study of 540 Swedish adults 20–70 years of age has revealed that three variables – smoking, greater age and higher mean plaque levels – were potential risk factors for severe periodontitis.[15] The risk for periodontitis is considerably greater for tobacco users, with estimated ratios in the range of 2.5–7.0 or even higher for smokers as compared with nonsmokers.[16] Even when the levels of plaque accumulation and gingival inflammation were not significantly different between smokers and nonsmokers, smokers exhibited an increase in prevalence as well as severity of destructive disease.[6,9] The relationship between smoking and periodontitis appears to be dose-dependent; the odds for more severe attachment loss range from 2.05 for light smokers to 4.75 in heavy smokers,[17] and there is a significant correlation between probing depth and smoking pack-years.[18] Furthermore, years of exposure to tobacco products have been shown to be a statistically significant risk factor for periodontal disease in 1156 community dwelling New England elders, regardless of other social and behavioral factors.[19]

In a study of 889 Spanish patients, gingival recession, probing depth, and clinical attachment level were significantly associated with smoking status. It was further noted that smoking one cigarette per day, up to 10, and up to 20, increased clinical attachment loss by 0.5%, 5% and 10%, respectively.

The data support those of Wouters *et al*.[20] who found significantly less alveolar bone in individuals smoking more than 5 g of tobacco per day than in those smoking between 1 and 5 g of tobacco per day, as well as those of Norderyd and Hugoson,[15] found that moderate to heavy smoking (greater than or equal to 10 cigarettes per day) was associated with severe periodontitis but light smoking (less than 10 cigarettes per day) was not. The response to surgical and nonsurgical periodontal treatment has been shown to be less favorable in smokers compared to nonsmokers in terms of probing depth reduction and clinical attachment gain, even in the presence of ongoing, effective supportive therapy.[5,21] Furthermore, in a group of refractory periodontitis patients, 90% of the subjects reported to be smokers.[22]

### 2. Role of smokeless tobacco products (cigar, pipe smoking) in periodontal disease

The relationship of the smokeless tobacco and oral carcinoma, white oral mucosal lesions has been well documented.[23] These lesions are commonly found in the areas of the mouth where smokeless tobacco products are placed and occur in 50-60% of smokeless tobacco users.[24–26] A clear relationship between smokeless tobacco use and generalized periodontal conditions has not been definitively demonstrated.[27] In general, localized attachment loss in the form of gingival recession occurs in 25 to 30% of smokeless tobacco users.[24–26] This attachment loss is most prevalent adjacent to mandibular buccal areas where smokeless tobacco products are commonly placed.[24] *In vitro* studies have demonstrated that smokeless tobacco extracts affect monocyte and oral keratinocyte production of inflammatory mediators which may play a role in the development of these localized tissue alterations. Relative to our knowledge of the effects of cigarettes and smokeless tobacco on the periodontium, there is less information regarding the periodontal effects of cigar and pipe smoking.[28–31] In the NHANES I survey conducted between 1971 and 1974, periodontal index scores did not vary among pipe, cigar and cigarette smokers.

At the baseline dental examination of the Veterans Administration Dental Longitudinal Study, which began in 1968, 862 male subjects were classified as cigarette smokers, pipe/cigar smoker or non smokers.[28] At the six-year follow-up, it was concluded that the periodontal status of the cigar/ pipe group was intermediate between that of the cigarette and nonsmoking groups.

The most recent data from this cohort included 690 men who returned for examination at least once over a 23-year period.[30] Compared to non smokers, smokers of cigars had a 1.3 fold risk of tooth loss, and pipe and cigarette smoker each had a 1.6 fold risk. This study separated pipe and cigarette smokers and included only men who smoked cigars, pipes or cigarettes exclusively during the follow up period. This factor as well as the longer duration of follow-up may explain this report’s finding of a greater negative periodontal impact of cigar and pipe smoking as compared to the earlier studies.

Regardless of the form of usage, all tobacco products contain hazardous chemicals with over 4000 known constituents. These include carbon monoxide, hydrogen cyanide, reactive oxidizing radicals, a high number of carcinogens and the main psychoactive and addictive molecule- nicotine.[32] Nicotine is a major and most pharmacologically active agent in tobacco. Possibly a major contributing factor for almost all the deleterious effects associated with tobacco.

## NICOTINE (DRUG REVIEW)

### History and name

Nicotine is named after the tobacco plant *nicotine tobaccum*, which in turn is named after Jean Nicot, French ambassador in Portugal who sent tobacco and seeds from Brazil to Paris in 1560 and promoted their medicinal use.[33] For thousands of years, people have smoked or chewed the leaves of tobacco plant. As early as 1600s, the people speculated that there might be a link between diseases like cancer and tobacco use.

### Chemistry

Nicotine is one of the few natural alkaloids. It is a colorless, volatile base (pKa = 8.5) that turns brown and acquires the odor of tobacco on exposure to air. Nicotine is hygroscopic, oily liquid that is miscible in water in its base form. As a nitrogenous base, it forms salt with acids that are usually solid and water soluble. Nicotine easily penetrates the skin. Nicotine burns at temperature below its boiling point and vapors will burn at 308k in air despite a low vapor pressure. Because of this, most of the nicotine is burned when a cigarette is smoked. However enough is inhaled to provide the desired effects.

### Pharmacological actions

The complex and often unpredictable changes that occur in the body after administration of nicotine are due to its actions on the neuroeffector and chemosensitive sites but also to the fact that the alkaloid can stimulate and desensitize receptors.

### Peripheral nervous system

The major action of nicotine consists initially of transient stimulation and subsequently of a more persistent depression of all autonomic ganglia. Nicotine also possesses a biphasic action on the adrenal medulla: small doses evoke the discharge of catecholamines, and larger doses prevent their release in response to splanchnic nerve stimulation. Nicotine, like Acetylcholine is known to stimulate a number of sensory receptors.

### Central nervous system

Nicotine markedly stimulates the CNS. Low doses produce weak analgesia; with higher doses tremors leading to convulsions at toxic doses are evident. The excitation of respiration is a prominent action of nicotine. Nicotine induces vomiting by both central and peripheral actions. The primary sites of action of nicotine in the CNS are prejunctional, causing the release of other neurotransmitters. Accordingly, the stimulatory and pleasure-reward actions of nicotine appear to result from release of excitatory aminoacids, dopamine, and other biogenic amines from various CNS centers.

### Cardiovascular system

The cardiovascular responses to nicotine are due to stimulation of sympathetic ganglia and the adrenal medulla, together with the discharge of catecholamines from sympathetic nerve endings. Also contributing to the sympathomimetic response to nicotine in the activation of chemoreceptors of the aortic and carotid bodies, this reflexly results in vasoconstriction, tachycardia, and elevated blood pressure.

### Gastrointestinal tract

The combined activation of parasympathetic ganglia and cholinergic nerve endings by nicotine results in increased tone and motor activity of the bowel.

### Exocrine glands

Nicotine causes an initial stimulation of salivary and bronchial secretions that is followed by inhibition.

### Absorption, fate and excretion

As nicotine enters the body, it is distributed quickly through the blood stream and can cross the blood brain barrier. It takes about seven seconds for the substance to reach the brain when inhaled, which is faster than the intravenous infusion. Nicotine in tobacco smoke from most cigarettes is not well absorbed from oral mucosa because nicotine is in an ionized form as a result of the pH (5.5) in contrast cigar and pipe smoke is more alkaline pH (8.5), which allows good absorption of unionized nicotine through the buccal mucosa.[34] Nicotine is readily absorbed from the respiratory tract, buccal membranes, and skin. Being a relatively strong base, its absorption from the stomach is limited, and intestinal absorption is far more efficient. Nicotine in chewing tobacco, because it is more slowly absorbed than inhaled nicotine, has a longer duration of effect. The average cigarette contains 6 to 11 mg of nicotine and delivers about 1 to 3 mg of nicotine systemically to the smoker; bioavailability can increase as much as threefold with intensity of puffing and technique of the smoker.[35,36]

Approximately 80 to 90% of nicotine is altered in the body, mainly in the liver but also in the kidney and lung. Cotinine is the major metabolite, with nicotine-1-N-oxide and 3-hydroxycotinine and conjugated metabolites found in lesser quantities. The half life of nicotine following inhalation or parenteral administration is about 2 hrs. The rate of urinary excretion of nicotine is dependent upon the pH of the urine. Nicotine is also excreted in the milk of lactating women who smoke; the milk of heavy smokers may contain 0.5 mg per liter.

### Acute nicotine poisoning

Poisoning from nicotine may occur from accidental ingestion of nicotine-containing insecticide sprays or in children from ingestion of tobacco products. The acutely fatal dose of nicotine for an adult is probably about 60 mg of the base. Smoking tobacco usually contains 1 to 2% nicotine. Apparently, the gastric absorption of nicotine from tobacco taken by mouth is delayed because of slowed gastric emptying, so that vomiting caused by the central effect of the initially absorbed fraction may remove much of the tobacco remaining in gastrointestinal tract.

The onset of symptoms of acute, severe nicotine poisoning is rapid; they include nausea, salivation, abdominal pain, vomiting, diarrhea, cold sweat, headache, dizziness, disturbed hearing and vision, mental confusion, and marked weakness. Faintness and prostration ensue; the blood pressure falls; breathing is difficult; the pulse is weak, rapid, and irregular, and collapse may be followed by terminal convulsions. Death may result within a few minutes from respiratory failure.[37]

### Therapy

Vomiting should be induced with syrup of ipecac or gastric lavage should be performed. Alkaline solutions should be avoided. Slurry of active charcoal is then passed through the tube and left in the stomach. Respiratory assistance and treatment of shock is necessary.

## THERAPEUTIC USES

1. Used in addicts as a replacement therapy to make them free of nicotine dependence.
2. Risk of ulcerative colitis has been shown to be decreased in smokers in dose dependant manner.
3. Proposed to interfere with development of Kaposi’s sarcoma and breast cancer in women carrying breast cancer (BRCA) gene, pre eclampsia, and atopic disorders such as allergic asthma owing to its anti-inflammatory action.
4. Use to help adults suffering from autosomal dominant nocturnal frontal lobe epilepsy.
5. A few of its metabolites are being researched for their potential role in a number of disorders including schizophrenia and Parkinson’s disease.
6. The therapeutic use of nicotine as a mean of appetite control and weight loss is anecdotally supported by many ex-smokers who claim to put on weight after quitting.

### Hallmarks of nicotine addiction

Compulsive use, use despite harmful effects, pleasant (euphoric) effects, difficulty in quitting or controlling use, recurrent drug cravings, tolerance, physical dependence, relapse following abstinence.

### Factors contributing to the negative impact of smoking on the periodontium

Various factors contribute to the deleterious periodontal effects of smoking, including alterations in both microbial and host response factors [Table 2].

**Table 2:** Proposed mechanisms for negative periodontal effects of smoking

Vascular alterations
Altered neutrophill function
Decreased IgG production
Decreased lymphocyte proliferation
Increased prevalence of periopathogens
Difficulty in eliminating pathogens by mechanical therapy
Altered fibroblast attachment and function
Negative local effects on cytokine and growth factor production

IgG - Immunoglobulin G

### Smoking and micro flora

There are conflicting reports on the effects of smoking on micro flora, with some studies reporting no difference in the prevalence of subgingival bacteria associated with periodontitis. However, data from the large Erie County study showed that the proportions of subjects positive for *Aggregatibacter actinomycetamcomitans.gen.nov*,[38] *Porphyromonas gingivalis*, and *Tannerella forsythia*[39] were higher among smokers,[40] and there are other reports of a higher prevalence of certain organisms in smokers.[41–43] Furthermore, increased counts of exogenous flora (*Escherichia coli* and *Candida albicans*) have been reported in smokers.

### Smoking and the host response

Numerous functions of oral or peripheral neutrophils are negatively affected by smoking or nicotine exposure, including phagocytosis,,[44] superoxide and hydrogen peroxide generation[45,46] integrin expression[47] and protease inhibitor production.[48] The immune response is also impaired by smoking. Alterations in gingival crevicular fluid[49–51] and peripheral blood mononuclear cell[52] levels of various cytokines in smokers, tipping the balance in favor of tissue breakdown, have been noted. Smoking decreases salivary IgA[53] and serum IgG,[54] and specifically reduces IgG2 to *Aggregatibacter actinomycetamcomitans.gen.nov*.[55]

*The* ability of tobacco products to decrease the proliferative capacity of T and B lymphocytes might contribute to this diminished production of protective antibodies.[56] Nicotine can suppress the osteoblast proliferation while stimulating the alkaline phosphatase activity.

### Local effects of nicotine

The oral tissues of smokers are exposed to high nicotine concentrations that negatively affect local cell populations. Gingival crevicular fluid nicotine concentrations can be up to nearly 300 times[47] that of nicotine plasma concentrations in smokers (20 ng/ml).[57] The vasoconstrictive properties of nicotine are hypothesized to impair gingival blood flow; however, studies that have examined the effects of smoking on gingival blood flow in humans have shown either no change or increased flow as measured by laser Doppler flowmetry.[58–60] This may be due to smoking-induced elevation in blood pressure, which overcomes any vasoconstrictive effects of smoking.[60] Smoking has been shown to impair revascularization during soft[61] and hard tissue wound healing,[62] which is critical for periodontal plastic, regenerative, and implant procedures. Nicotine binds to root surface in smokers,[63] and *In vitro* studies show it can alter fibroblast attachment[64,65] and integrin expression,[66] and decrease collagen production while increasing collagenase production.[67] Root surfaces of teeth extracted from smokers show reduced periodontal ligament (PDL) fibroblast attachment as compared to those from non-smokers.[68] Cultured gingival keratinocytes[69] and fibroblasts[70] exposed to nicotine produce higher amounts of the proinflammatory cytokines IL-1 and IL-6, respectively.

Furthermore, there is evidence of a synergistic effect on inflammatory mediator production when bacterial lipopolysaccharide is combined with nicotine.[70,71] Animal studies have shown that local nicotine delivery negatively impacts bone healing,[72] which may be related to inhibited expression of various growth factors[73] and delayed revascularization.[62] These findings might help explain the diminished treatment response to surgical periodontal procedures, especially those involving tissue regeneration.

### Impact of smoking cessation on periodontal status and treatment outcome

While smoking cessation does not reverse the past effects of smoking, there is abundant evidence that the rate of bone and attachment loss slows after patients quit smoking, and that their disease severity is intermediate to that of current and non smokers.[74–80] It is encouraging to note that former smokers respond to non-surgical and surgical therapy in a manner similar to never smokers.[81,82]

In fact, among patients who had quit smoking one year or more prior to scaling and root planing, there was no relationship between the number of years since cessation and changes in probing depth or clinical attachment levels.[81] Similarly, implant success rates for past smokers are similar to those for never smokers.[83] According to Bain,[84] if patients quit smoking one week before and eight weeks after implant placement, early implant failures were similar to non-smokers.

### Role of dental health professionals in tobacco cessation

Dentistry has a strong history of commitment to preventive education as a routine part of patient treatment. The practice of periodontics offers multiple opportunities for interaction with patients: during active treatment and especially in the ongoing long-term maintenance phase. In a survey of general dentists, 65% claim to advise most or all of their patients who smoke to quit, but few provide cessation counseling.[85] There are many possible approaches to tobacco use intervention in the dental office, ranging from brief interventions to comprehensive cessation programs involving the entire office staff. Nicotine dependence is classified as a chemical addiction by the American Psychiatric Association in the Diagnostic and Statistical Manual of Mental Disorders 1994 (DSM-IV).[86] Although tobacco use is a learned behavior with social implications and has characteristics of a habit, the main motivation behind continued use is relief of withdrawal symptoms. The symptoms can include irritability, anxiety, decreased heart rate, increased appetite, food cravings, restlessness, and difficulty concentrating.[87]

A systematic approach that combines behavioral counseling with pharmacotherapy has been shown to achieve the highest rates of cessation, although each is also effective alone.

## TOBACCO INTERVENTION MODELS FOR DENTAL PRACTICE

### Brief intervention program

Inadequate information about treatment options, time constraints, lack of compensation, and unrealistic expectations are common reasons that prevent practitioners from offering these services.[88] In offices where time is an issue or where practitioners lack confidence in pursuing more comprehensive programs, a useful model for brief intervention that uses a five step approach is recommended by the Agency for Health Care Research and Quality. The program is known as the five A’s for smoking cessation. It includes: ask - systematically identify the tobacco use status of all patients; advise - strongly advise all who use tobacco products to stop; assess - evaluate patient’s willingness to quit; assist - offer assistance in quitting; and arrange following up on the patient’s cessation efforts, especially early in the process.[89] The emphasis in this brief intervention is to offer information, encouragement, and support to patients, and to provide information about resources that may help the patient become tobacco free. All smokers benefit from the advice of a trusted health professional; in up to 10% of cases, the simple statement of encouragement to stop smoking will cause the patient to give up smoking.[90]

### Comprehensive intervention program

A model for a comprehensive program in the dental office includes using the five As and expanding the scope of intervention. Identification of an office coordinator for tobacco cessation activities is the first step in involving the entire office staff. The most ideal person to implement this program in the dental office is a dental hygienist. A cessation program tailored to the patient’s needs should be offered, one that ideally combines counseling, pharmacological therapy using both nicotine replacement and other medications, and supportive follow-up.[91]

#### Expanding the five as for comprehensive intervention

Ask: Identification of the patient’s tobacco use status (current, former, or never) is the first step in all interventions. The addition of tobacco use status to the traditional vital signs has been suggested as a way to initially assess and update this information,[92] and a question regarding tobacco use should be a part of the health questionnaire used in the dental office. The Fagerström test is scored based on answers to questions about timing of the first cigarette smoked in the day, difficulty in not smoking in forbidden areas, most important cigarette during the day, number of cigarettes smoked per day, timing of most intense smoking, and smoking when ill. Higher scores indicate more addicted smokers.[93]

Advise: A good time to advise is after the periodontal examination has been completed and during a review of the etiologic factors involved in periodontal diseases. Facts regarding the strength of smoking as a risk factor for disease, its impact on treatment, and the positive impact of cessation are statements that can be included in a manner that is informative and not judgmental. The patient’s responses during this discussion will provide insight into their interest in cessation and level of readiness for cessation.

Assess: The next step is to identify the patient’s interest and readiness to attempt tobacco cessation. A transtheoretical model for readiness to change is useful for evaluation of addictive behaviors, and is used frequently for tobacco cessation counseling. It is a five-stage model that identifies behavior change as a process involving movement through these categories. This model is described as a spiral, and includes precontemplation, contemplation, preparation, action, and maintenance.[94] The most effective interventions can occur when the patient is in the preparation or action stage, but all patients can benefit from appropriate counseling based on their current stage of change. An intervention should be considered successful if some movement is made in the stages of change model, even if it does not lead immediately to cessation. Patients in the preparation stage are willing to attempt cessation and are ready for behavioral intervention and pharmacological therapy. When patients are in the action or maintenance stages, relapse prevention is critical to continued abstinence.[95] Patients may cycle through these stages multiple times before they achieve success in becoming tobacco free.[96]

Assist: A style of behavior change counseling that has grown in popularity is motivational interviewing, a method that helps patients explore and resolve ambivalence about changing behaviors.[97] There are many other alternatives in behavioral therapy that have been studied and used successfully in tobacco use counseling, for example stimulus control and hypnosis. The best use of behavioral intervention might be in combination with pharmacological treatment.

Arrange: If the patient makes a commitment to smoking cessation, follow-up from the office is critical. Methods of maintaining contact with the patient can range from appointments for office visits for monitoring and continued counseling, to letters or telephone calls confirming quit dates and encouraging follow through with cessation. The most difficult time for patients is usually during the first week of cessation. Research shows that cessation rates are positively influenced by follow-up contact.[98]

### Pharmacotherapy

The use of pharmacotherapy in tobacco cessation began in the 1980s, when nicotine replacement therapies were introduced. The U.S. Food and Drug Administration (FDA) currently approves nicotine chewing gums, nicotine lozenges, nicotine patches, nicotine nasal sprays, and nicotine inhalers for use in patients who are attempting cessation.

The patches, chewing gums, and lozenges are available as over-the-counter products; the inhaler and nasal spray require prescriptions. Nicotine replacement products act as nicotine delivery systems in lieu of tobacco and can decrease withdrawal symptoms.[99–101] One non-nicotine medication, sustained-release Bupropion, is also approved for tobacco cessation pharmacotherapy. These medications have been proven safe and effective, and have been extensively studied alone, in combination, and as an adjunct to behavioral therapy.[100–104] Barring complications, all patients attempting cessation should be treated with at least one form of pharmacotherapy. In general, the addition of medication to behavioral therapy doubles cessation rates.[105] Nicotine replacement products and sustained release Bupropion are considered first-line therapies. Clonidine and nortriptyline are second-line pharmacotherapies that have been studied for cessation therapy, but have more side effects and are not approved at this time by the FDA for use in tobacco cessation.

### Indications for use of pharmacotherapy

In creating a personalized treatment plan for tobacco cessation, the patient’s health history is important. The presence of other health problems may influence the approach that is used, and consultation and cooperative treatment in conjunction with the patient’s physician are always appropriate. The use of nicotine replacement products should be related to the patient’s current nicotine exposure and to past experiences with cessation. Estimation of the daily amount of nicotine obtained from cigarettes can be calculated with accurate information about the daily number of cigarettes smoked. A single cigarette has about 3 mg of nicotine. On average, a smoker gets approximately 1 mg of nicotine from a cigarette leisurely smoked over about a five-minute period. However, smokers who smoke more rapidly and inhale deeply can get up to 3 mg of nicotine from a cigarette.[106] The ability to subconsciously titrate nicotine dosages is one of the reasons that patients who claim they have decreased the number of cigarettes smoked per day are still able to maintain the same blood levels of nicotine with fewer cigarettes, and successfully prevent withdrawal symptoms.

## CONCLUSION

Clinical and epidemiological studies support the concept that tobacco use is an important variable affecting the prevalence and progression of periodontal diseases such as adult periodontitis, refractory periodontitis, generalized early-onset periodontitis, and ANUG. In studies in which plaque levels were adjusted between smokers and non-smokers, greater probing depths, clinical attachment loss, and bone loss have been reported in smokers. Several studies have demonstrated that the severity of periodontal disease appears to be related to the duration of tobacco use, smoking status, and amount of daily tobacco intake. Studies comparing therapy response in former smokers, current smokers, and never smokers demonstrate that former smokers respond to periodontal therapy in a manner to should advise patients of tobacco’s negative health effects as well as the benefits of quitting tobacco use, and tobacco cessation counseling should be part of the armamentarium of the dental office.
